# Antimicrobial Photodynamic Inactivation Mediated by Tetracyclines in Vitro and in Vivo: Photochemical Mechanisms and Potentiation by Potassium Iodide

**DOI:** 10.1038/s41598-018-35594-y

**Published:** 2018-11-20

**Authors:** Weijun Xuan, Ya He, Liyi Huang, Ying-Ying Huang, Brijesh Bhayana, Liyan Xi, Jeffrey A. Gelfand, Michael R. Hamblin

**Affiliations:** 1 Department of Otorhinolaryngology, Head and Neck Surgery, First Clinical Medical College and Hospital, Guangxi University of Chinese Medicine, Nanning, China; 2 Wellman Center for Photomedicine, Massachusetts General Hospital, Boston, MA USA; 3 Department of Dermatology, Harvard Medical School, Boston, MA USA; 4 Department of Dermatology, Sun Yat-sen Memorial Hospital, Sun Yat-sen University, Guangzhou, Guangdong China; 5 Department of Infectious Diseases, First Affiliated Hospital, Guangxi Medical University, Nanning, China; 6 Dermatology Hospital of Southern Medical University, Guangzhou, Guangdong China; 7 Department of Medicine, Massachusetts General Hospital, Boston, MA USA; 8 Harvard-MIT Division of Health Sciences and Technology, Cambridge, MA USA

## Abstract

Tetracyclines (including demeclocycline, DMCT, or doxycycline, DOTC) represent a class of dual-action antibacterial compounds, which can act as antibiotics in the dark, and also as photosensitizers under illumination with blue or UVA light. It is known that tetracyclines are taken up inside bacterial cells where they bind to ribosomes. In the present study, we investigated the photochemical mechanism: Type 1 (hydroxyl radicals); Type 2 (singlet oxygen); or Type 3 (oxygen independent). Moreover, we asked whether addition of potassium iodide (KI) could potentiate the aPDI activity of tetracyclines. High concentrations of KI (200–400 mM) strongly potentiated (up to 5 logs of extra killing) light-mediated killing of Gram-negative *Escherichia coli* or Gram-positive MRSA (although the latter was somewhat less susceptible). KI potentiation was still apparent after a washing step showing that the iodide could penetrate the *E*. *coli* cells where the tetracycline had bound. When cells were added to the tetracycline + KI mixture after light, killing was observed in the case of *E*. *coli* showing formation of free molecular iodine. Addition of azide quenched the formation of iodine but not hydrogen peroxide. DMCT but not DOTC iodinated tyrosine. Both *E*. *coli* and MRSA could be killed by tetracyclines plus light in the absence of oxygen and this killing was not quenched by azide. A mouse model of a superficial wound infection caused by bioluminescent *E*. *coli* could be treated by topical application of DMCT and blue light and bacterial regrowth did not occur owing to the continued anti biotic activity of the tetracycline.

## Introduction

Antimicrobial photodynamic inactivation (aPDI) is a new approach to killing infectious pathogens, that is independent of existing antibiotic resistance status, and is not thought likely to cause any resistance to develop itself^1^. Research in this area has been driven by growing international concern about the seemingly unstoppable rise of multi-drug resistance amongst bacteria and other pathogenic microorganisms, that was highlighted in the O’Neill report^2^. aPDI is based upon excitation of a dye molecule (called a photosensitizer, PS) by visible light. The PS forms a long-lived triplet state, that can react with oxygen to produce reactive oxygen species (ROS) including singlet oxygen and hydroxyl radicals^3^. These ROS can attack critical biomolecules (lipids, proteins, nucleic acids) producing cell lysis of the microorganisms and death. Selectivity for microbial cells (compared to host mammalian cells) is provided by the selection of a suitable cationic PS structure designed to preferentially bind to microbial cells that tend to be negatively charged. Additional selectivity is obtained by local administration of the PS into the infected area, confining the light only to the infected area, and the use of a short-drug-light interval, because uptake by mammalian cells is slow while binding to bacteria is rapid.

There has been a wide variety of PS structures that have been reported to be effective in aPDI^4,5^, including well established dyes such as the phenothiazinium salt methylene blue^6^, xanthenes such as Rose Bengal^7^, and carbocyanines such as indocyanine green^8^. Moreover a host of newer cationic derivatives of tetrapyrrole structures (porphyrins^9^, phthalocyanines^10^, and bacteriochlorins^11^) have been reported to have very high activities. One class of chemical structures that has not been much investigated however, is that of the antibiotics themselves. This is somewhat surprising, as phototoxicity has long been known to be one of the most troubling side-effects of antibiotics that are clinically administered for many infectious diseases^12^.

One of the most well-known examples of phototoxic antibiotics, is the class of tetracyclines in general, and doxycycline, demeclocycine, and tetracycline in particular^13–15^. Tetracyclines are a class of antibiotics first isolated in 1948^16^, that are bacteriostatic in nature and function by reversibly inhibiting the bacterial ribosome 30S subunit. Tetracyclines can gain entry to bacterial cells via the OmpF and OmpC porin channels^17^. Once inside the periplasm the uncharged tetracycline can diffuse through the lipid bilayer of the cytoplasmic membrane to reach the ribosomes^18^. Therefore tetracyclines have fundamentally different mechanisms of uptake and subcellular targeting compared to the vast majority of alternative antibacterial PS, that rely on cationic charge and the self-promoted uptake pathway^19^.

The first report that tetracyclines could act as antibacterial PS was published as long ago as 1987 by Martin *et al*.^20^. Since then there have only been two more reports about carrying out aPDI with tetracyclines that we can trace^21,22^. We recently investigated whether tetracyclines could function as dual-action light activated antimicrobial compounds using either UVA (360 nm) or blue (415 nm) light^23^. We found that in contrast to well-established PS such as MB, that was only effective when incubated with the bacteria in PBS and had no effect in rich growth medium such as Brain Heart Infusion Broth, tetracyclines localized inside the bacterial cells and were not affected by the protein present in the medium. When tetracyclines were used to photoinactivate a few logs of bacterial CFUs, the remaining antibiotic was sufficient to prevent any regrowth in media after the end of light delivery, which was not the case with MB. Moreover, we were able to show that the MICs of tetracyclines measured by a broth microplate dilution assay, were lower (more effective) when measured under 0.5 mW/cm^2^ of continuous blue light than when measured in the dark.

We recently reported that addition of potassium iodide (KI) could potentiate aPDI using a range of different PS. When KI was added to MB and excited with red light, the killing of Gram-positive bacteria, Gram-negative bacteria and fungi was significantly enhanced^24^. We next showed that addition of KI could potentiate aPDI by functionalized fullerenes^25^ and photocatalytic titanium dioxide nanoparticles^26^. Since MB, fullerenes and TiO_2_ photocatalysis are able to catalyze photoinduced electron transfer, we hypothesized that this was the mechanism of action. However, we then discovered that KI could also potentiate aPDI carried out by different PS that are Type II (i.e. they can produce singlet oxygen) such as Photofrin^27^ and Rose Bengal^28^. We now hypothesized that an addition reaction of singlet oxygen to iodide anion can form peroxyiodide, which subsequently decomposes into molecular iodine and hydrogen peroxide^27^. The bacterial killing is therefore probably caused by a mixture of extracellular free iodine (I_2_/I_3_^−^), and reactive iodine radicals (I^•^/I^•^_2_) depending on the PS structure and its degree of binding to the microbial cells.

Therefore, the goal of the present study was to ask whether addition of KI could also potentiate aPDI mediated by tetracyclines in Gram-positive (MRSA) and Gram-negative (*Escherichia coli*) bacteria. We conducted some studies designed to elucidate the photochemical mechanisms involved, and explored the possibility of photoinactivation of bacteria in the absence of oxygen. Finally, we tested the ability of PDT mediated by DMCT and blue light to treat a superficial wound infection in mice caused by a bioluminescent strain of pathogenic *E*. *coli*.

## Materials and Methods

### Chemicals

Demeclocycline (DMCT), doxycycline (DOTC), potassium iodide, sodium azide, were from Sigma-Aldrich (St. Louis, MO). Stock solutions of tetracyclines were freshly prepared each day in distilled H_2_O (dH_2_O) at 10 mM (around 5 mg/mL) and for salts at 5 M. Phosphate-buffered saline (PBS) for microbial cell suspension and serial dilutions, brain-heart infusion broth (BHI), and agar for bacterial growth were purchased from Fisher Scientific (Waltham, MA). Starch was from RICCA Chemical Company (Arlington, TX). Amplex red hydrogen peroxide/peroxidase assay kit, singlet oxygen sensor green (SOSG) and hydroxyphenyl-fluorescein (HPF) were purchased from Invitrogen (Carlsbad, CA).

### Light sources

A 365-nm UVA light-emitting diode (LED) light source (Larson Electronics LLC, Kemp, TX), a 415-nm blue light LED light source (Omnilux Clear-U, Glen Ellen, CA) were used for *in vitro* experiments^23^. For light intensity measurements, a model IL-1700 research radiometer-photometer (International Light, Inc., Newburyport, MA) was used for the UVA light and a model DMM 199 power meter (Coherent, Santa Clara, CA) was used for blue light. Both the blue light, and UVA sources could deliver a light spot covering six wells of a 24-well plate at an irradiance of 12 mW/cm^2^ (1 J/cm^2^ delivered in 1.4 min). We used a prototype light-emitting diode (LED) (Vielight, Inc., Toronto, Canada) with peak emission at 415 nm and full-width half maximum of 20 nm for *in vivo* experiments. The LED was mounted on a heat sink to prevent any thermal effects on the irradiated tissue. The irradiance on the mouse surface was 70 mW/cm^2^. Varying fluences were delivered by varying the irradiation time.

### Bacterial strains and culture conditions

The *Escherichia coli* UTI 89^29^ and methicillin-resistant *Staphylococcus aureus* (MRSA) US300 were grown in liquid BHI medium with shaking at 120 rpm at 37 °C overnight to reach stationary phase. One mL of this suspension was then refreshed in fresh BHI to mid-log phase. Cell numbers were estimated by measuring the OD at 600 nm (OD of 0.6 = 10^8^ CFU/mL). The bacterial suspension was spun down, washed, and resuspended in PBS for the experiments.

### aPDI studies

Suspensions of bacteria (10^8^ cells/mL) were incubated in the dark at 25 C for 30 min with the stated concentration of tetracycline with the addition of a range of KI concentrations varying between 0 and 400 mM in pH 7.4 PBS. An aliquot of 100 μL was used as the dark control (DC) from each sample; another aliquot (200 µL) was transferred to a 96-well plate and illuminated from the top at room temperature with 10 J/cm^2^ of UVA light or blue light. This was called the “in format”. For the “spin format”, centrifugation (5 min, 4,000 rpm) of 1 mL aliquots was used to remove the excess of tetracycline that was not taken up by the bacterial cells, the pellet was then resuspended in PBS and the KI was added and illumination took place^30^. For the “after format”, tetracycline and KI were mixed in PBS without cells and illuminated. Immediately after the light dose was completed, the cell suspension was added to the solution and mixed. At the completion of illumination (or dark incubation), aliquots (100 µL) were taken from each well to determine CFU. The aliquots were serially tenfold diluted in PBS to give dilutions of 10^−1^ to 10^−5^ times in addition to the original concentration and 10 µL aliquots of each of the dilutions were streaked horizontally on square BHI agar plates as described by Jett *et al*.^31^. Plates were streaked in triplicate and incubated for 12–18 h at 37 °C in the dark to allow colony formation. Each experiment was performed at least three times.

Suspensions of *E*. *coli* (10^8^ cells/mL) were incubated in the dark at room temperature for 30 min with 50 µM tetracyclines plus 400 mM KI with or without the addition of 50 mM NaN_3,_ and were then illuminated with 10 J/cm^2^ of 415 nm blue light. The aliquots were serially tenfold diluted as before. Each experiment was performed at least three independent times.

A control group of cells treated with UVA light or blue light alone (no tetracycline added) showed the same number of CFU as the absolute control (data not shown). Survival fractions were routinely expressed as ratios of CFU of cells treated with PDT to control CFUs.

### Amplex red assay for hydrogen peroxide

The Amplex red hydrogen peroxide/peroxidase assay kit was used to detect the production of H_2_O_2_ during tetracycline mediated PDT^27^. The colorless probe Amplex red (10-acetyl-3,7-dihydroxy-phenoxazine) reacts with H_2_O_2_ in the presence of peroxidase and forms the fluorescent compound, resorufin (7-hydroxy-3H-phenoxazin-3-one)^32^. The detection process after PDT was according to manufacturer’s instructions. The reaction systems contained tetracycline (50 µM) with or without addition of 400 mM KI. These were illuminated with increasing fluence of UVA or 415 nm light and aliquots were withdrawn and added to 50 μM Amplex Red reagent mixed with 0.1 U/mL horseradish peroxidase (HRP) in Krebs–Ringer phosphate. After 30 min incubation, a fluorescence microplate reader (excitation 530 nm and emission ~590 nm) was used to measure incremental fluorescence after each incremental fluence of light. Controls were (1) tetracycline + KI in dark, (2) KI + light, and (3) Amplex red reagent alone. Each experiment was performed at least three times.

### Iodine starch test

50 µM tetracyclines plus 400 mM KI with or without 50 mM sodium azide were illuminated with increasing fluences of UVA or 415 nm light, aliquots (50 μL) were withdrawn after different fluences, and added to starch indicator solution (50 μL)^27^. A microplate reader (absorbance 610 nm) was used to measure incremental absorbance after an incremental fluence of 415 nm light was delivered. Controls were (1) tetracyclines + KI in dark, (2) KI + light, (3) tetracyclines + light. Each experiment was performed at least three times.

### Activation of SOSG and HPF

Singlet oxygen sensor green (SOSG) is an anthracene derivative of fluorescein which is activated by addition of singlet oxygen to the anthracene moiety thus releasing the fluorescent dye fluorescein^33^. Hydroxyphenyl-fluorescein (HPF) is activated by attack of hydroxyl radicals onto the phenol ring thus releasing fluorescein^34^. Although neither probe is 100% specific by itself, when used together they are useful for identifying the ROS involved^35^.

Cell-free experiments were performed in 96-well plates. Tetracyclines were used at 10 µM in PBS, and the fluorescent probes, SOSG or (Molecular Probes, Invitrogen, USA) were added to each well at a final concentration of 10 μM. Each experimental group contained four wells that were illuminated simultaneously with UVA light in sequential doses of 0–1.0 J/cm^2^. UVA light does not activate the probes in the absence of tetracycline. A microplate spectrophotometer (Spectra Max M5, Molecular Devices) was used to measure fluorescence signals in the “slow kinetic” mode. The fluorescence excitation was 505 nm and emission was 525 nm. Each time after an increment of UVA light was delivered, the fluorescence was measured.

### Iodination of N-acetyl tyrosine ethyl ester

Sample solutions (total volume 400 μL) contained DMCT or DOTC (100 µM), KI (400 mM) and N-acetyl-L-tyrosine ethyl ester (10 mM) in PB buffer (pH 7.4, containing 10% methanol) were irradiated by UVA light (365 nm) for DOTC or with blue light (415 nm) for DMCT with magnetic stirring. An aliquot of solution (50 μL) was removed at different time point (30 mins, 60 mins, 120 mins) and centrifuged at 4000 rpm. It was necessary to use relatively large fluences of light in order to get enough product to allow measurement of the peak area. The supernatants were collected for the LC-MS analysis. The LC-MS analyses were performed on an Agilent 1260 LC system equipped with a triple-quad mass spectrometer. The LC conditions were: column: C18, 2.1 × 50 mm, 1.8 μm; elution gradient: solution A = acetonitrile, solution B = 10 mM ammonium acetate in water, 2% −> 100% of A over 6 min with a flow rate of 0.2 mL/min; ionization mode: negative; injection volume: 5 uL. The mass of the molecular ions of N-acetyl-3-iodo-L-tyrosine ethyl ester was 377^26^.

### Oxygen independent PDI

Suspensions of *E*. *coli* UTI 89 or MRSA (10^8^ cells/mL) plus 100 µM DMCT/DOTC (MRSA was only done with DMCT) with or without added 50 mM NaN_3_ were incubated in the dark at room temperature for 30 min, then illuminated with 10 J/cm^2^ of 415 nm blue light or UVA light. The aliquots were serially tenfold diluted as before. To remove oxygen, a mixture of cells, PS, and sodium azide (if required) contained in a quartz cuvette (Model 32Q10, Starna Cells Inc., Atascadero, CA), containing a magnetic stirrer and sealed with a rubber septum in the dark. The septum was pierced with a hollow needle connected to a N_2_/Ar line and samples were then bubbled with 75% N_2_/25% Ar gas at least for 30 min. The quartz cuvette allowed light to be delivered at 10 J/cm^2^ of 415 nm blue light or UVA light without admitting ambient air. Each cuvette was opened and serially diluted after 10 J/cm^2^ of 415 nm blue light or UVA light had been delivered. Each experiment was performed at least three independent times.

### *In vivo* studies

All animal experiments were approved (protocol 2005N000111) by the IACUC of Massachusetts General Hospital and met National Institutes of Health (NIH) guidelines.

### Bioluminescence imaging

The IVIS® Lumina Series III (PerkinElmer, Inc., Waltham, MA, USA) was used to carry out bioluminescence imaging on a daily basis until loss of the signal. Using the photon counting mode, an image was obtained by detection and integration of the individual photons emitted by the bacterial cells. Prior to imaging, mice were anesthetized by inhalation of isoflurane/oxygen mixture. Mice were then placed on a movable platform within the imaging chamber positioned directly under the camera. A grayscale reference image of each mouse was captured, followed by a bioluminescence image of the same region displayed in a false-color scale ranging from red (most intense) to blue (least intense) and superimposed on the grayscale image. The signal from the bioluminescence image was quantified as region of interest (ROI) with absolute calibrated data in photons s^−1^ cm^−2^ sr^−1^ using the IVIS software^36^.

### *E*. *coli* UTI 89 infection in mice

Adult female BALB/c mice 6–8 weeks-old weighing 18–21 g were obtained from Charles River Laboratories, MA, USA. Mice were given access to food and water *ad libitum*, and maintained on a 12-hour light/dark cycle at 21 °C. Mice were anesthetized by i.p. injection of ketamine/xylazine cocktail. They were shaved using an electric razor on the dorsal surface. A surgical scalpel was used to gently scrape the epidermis off an area of skin approximately 1 cm × 1 cm in order to create abrasion wounds. The depth of the wound was no more than the shallow dermis. After creating the wounds, a 50 μL aliquot of bacterial suspension containing 5 × 10^8^ CFU of *E*. *coli* UTI 89 in PBS was topically inoculated onto each defined area of the abrasion with a pipette tip. Bioluminescence images were taken immediately after the inoculation of bacteria to ensure that the amount of bacteria applied to each abrasion was consistent^25^.

### Mouse model of skin-abrasion infected *E*. *coli* UTI 89 and follow-up

Twenty two mice were randomly divided into 4 groups; these groups were designated as follows: (A) Infection control group: abrasion wounds were only infected with *E*. *coli* UTI 89 (n = 7 mice); (B) Dark control group: 500 µM DMCT, no light (n = 5 mice); (C) DMCT PDT group: 500 µM DMCT irradiated with 415 nm light (n = 5 mice); (D) 415 nm light-alone control group: irradiated with 415 nm blue light only, no DMCT (n = 5 mice).

The infected abrasion wounds were incubated for 60 min, a 50 μL aliquot of DMCT solution (500 μM) was added to the PDT-treated wound and also to dark controls at dark room temperature. Initially, 50 μL of the DMCT solution was added to the abrasions and incubated for 30 min to bind to and penetrate the bacteria. After 30 min the mice were imaged to quantify any dark toxicity of the DMCT to the bacteria. Then the wounds irradiated with 415 nm blue light to deliver 20, 40, 80 J/cm^2^ at an irradiance of 70 mW/cm^2^, follow by luminescence imaging and another addition 20 μL of the DMCT solution were performed after each aliquot of light dose. Sterile saline (0.5 mL intraperitoneally) was administered to support fluid balance during recovery. For (A), (B) and (D) group, luminescence imaging and another addition 20 μL of the DMCT [For (B) group] or PBS [For (A) and (D) group] were performed at the same different time points as PDT group. To record the time course of the extent of bacterial infection, the bacterial bioluminescence from mouse wounds was measured daily for 5 days after the wounds until the infections were cured (characterized by the disappearance of bacterial luminescence) or the wounds were healed. Finally the mice were euthanized.

## Results

### aPDI studies

**Figure 1:**
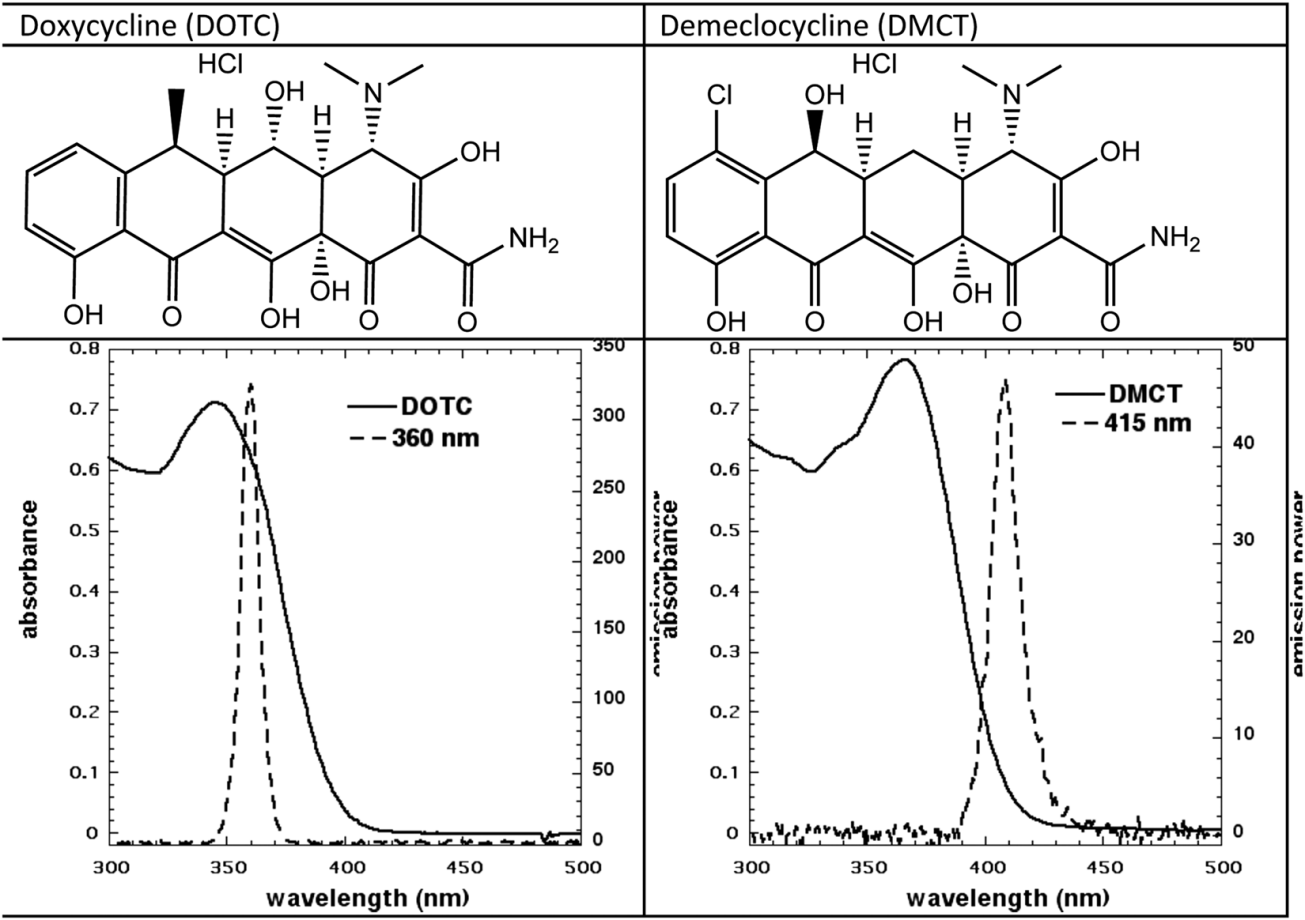
Structure and absorption spectra of DOTC and DMCT and emission spectra of UVA and blue light sources.

The structures, absorption spectra of the two tetracyclines, and the emission spectra of the respective UVA and blue light sources are shown in Fig. 1. It is apparent that although the overlap between the UVA light source and DOTC absorption is excellent, the overlap between DMCT and the blue light source is less than perfect. We continued to use blue light to excite DMCT because we had used this wavelength of light in our previous study^37^, and also because it was thought that UVA might be considered dangerous to use in a potential clinical application. It is important to ensure that the aqueous solutions of tetracyclines are prepared freshly each day before use. Even when stock solutions were stored in the dark at 4 C overnight they appeared to lose PDT activity.

**Figure 2:**
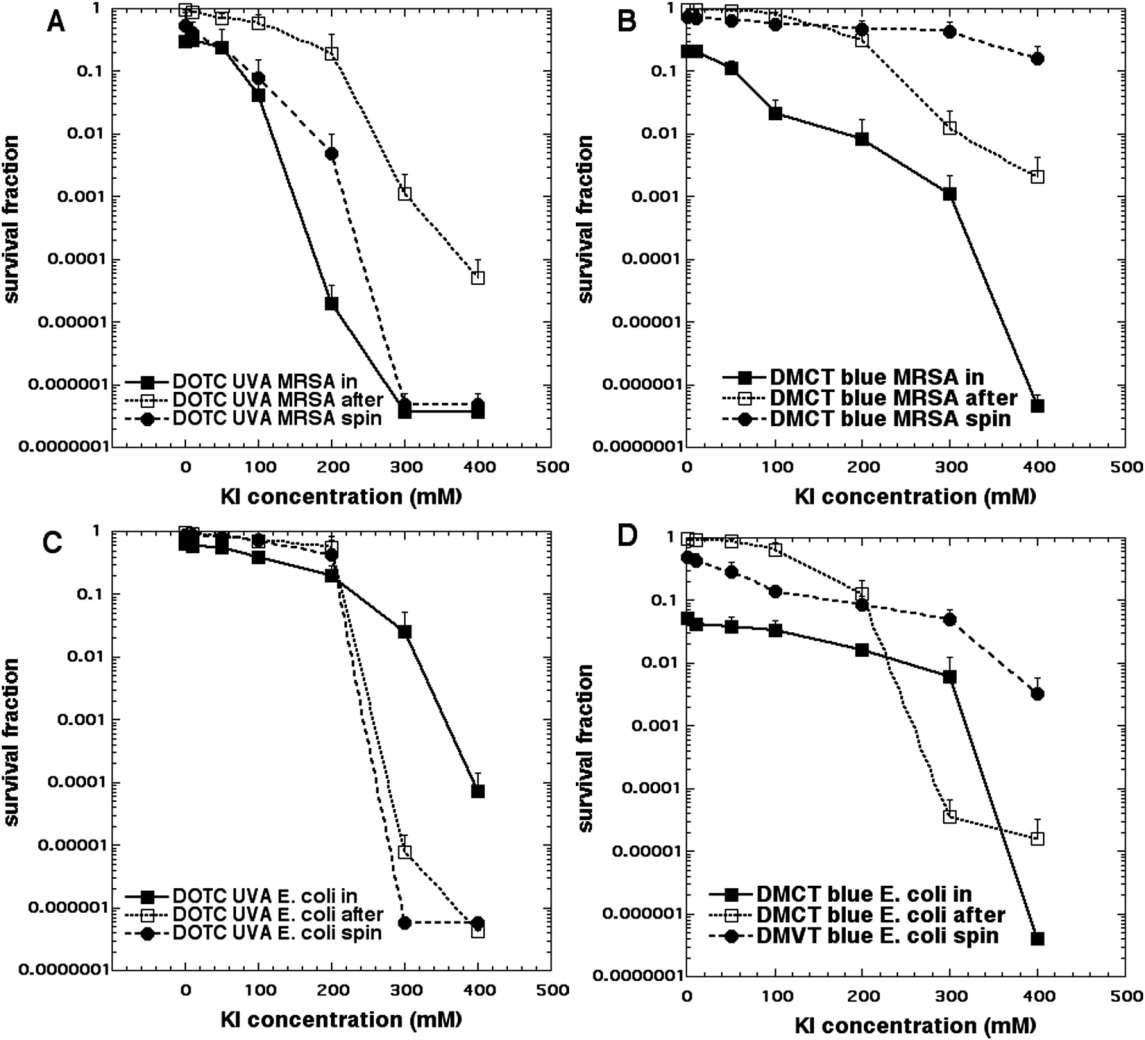
Potentiation of aPDI by addition of KI. Bacteria (10(8) CFU/mL), tetracyclines (3 µM), exposed to 10 J/cm^2^ of UVA or blue light with the addition of different concentrations of KI. Cells were either present during light (in format), centrifuged before addition of KI and light (spin format), or added after light (after format). Controls (light alone or light + KI) showed no killing (data not shown). (**A**) Gram-positive MRSA with DOTC excited by UVA; (**B**) MRSA with DMCT excited by blue light; (**C**) Gram-negative *E*. *coli* with DOTC excited by UVA light; (**D**) *E*. *coli* with DMCT excited by blue light.

We eventually decided to use a 3 µM solution of the fresh antibiotics as PS because the goal was to obtain only about 1 log of aPDI killing using the “in format”. We also tested bacterial killing in the “after format” where cells are added after light, and in the “spin format where cells are centrifuged after incubation with tetracycline and before addition of KI solution and light delivery. The main variable was the concentration of KI which we needed to increase all the way up to 400 mM in order to obtain the maximum potentiation. Figure 2 shows the results. Figure 2A shows MRSA incubated with 3 µM DOTC excited by 10 J/cm^2^ of UVA light which produced <1 log of killing, but which led to eradication (>6 logs killing) in both “in” and “spin” formats when the KI concentration reached up to 300 mM. The killing in the “after format” was less, but nevertheless 4 logs was obtained at a KI concentration of 400 mM. Figure 2B shows the results with MRSA and 3 µM DMCT excited with 10 J/cm^2^ of blue light. Eradication was achieved in the “in format” with 400 mM KI, and substantially lower killing (2.5 logs) using “after format” and hardly any killing with the “spin format”.

Figure 2C shows the analogous results with *E*. *coli* and 3 µM DOTC excited by 10 J/cm^2^ of UVA light. The highest potentiation was seen with the “spin format” with eradication being achieved with 300 mM KI, and then the “after format” where eradication was seen with 400 mM KI. The “in format” did not give as much potentiation with only 4 logs of killing seen with 400 mM KI. Figure 2D shows the effects of KI on aPDI of *E*. *coli* mediated by 3 µM DMCT and 10 J/cm^2^ of blue light. The “in format” gave eradication at 400 mM KI, while the “after format” was less effective, and the “spin format” only gave 2 logs of killing.

### Production of iodine

**Figure 3:**
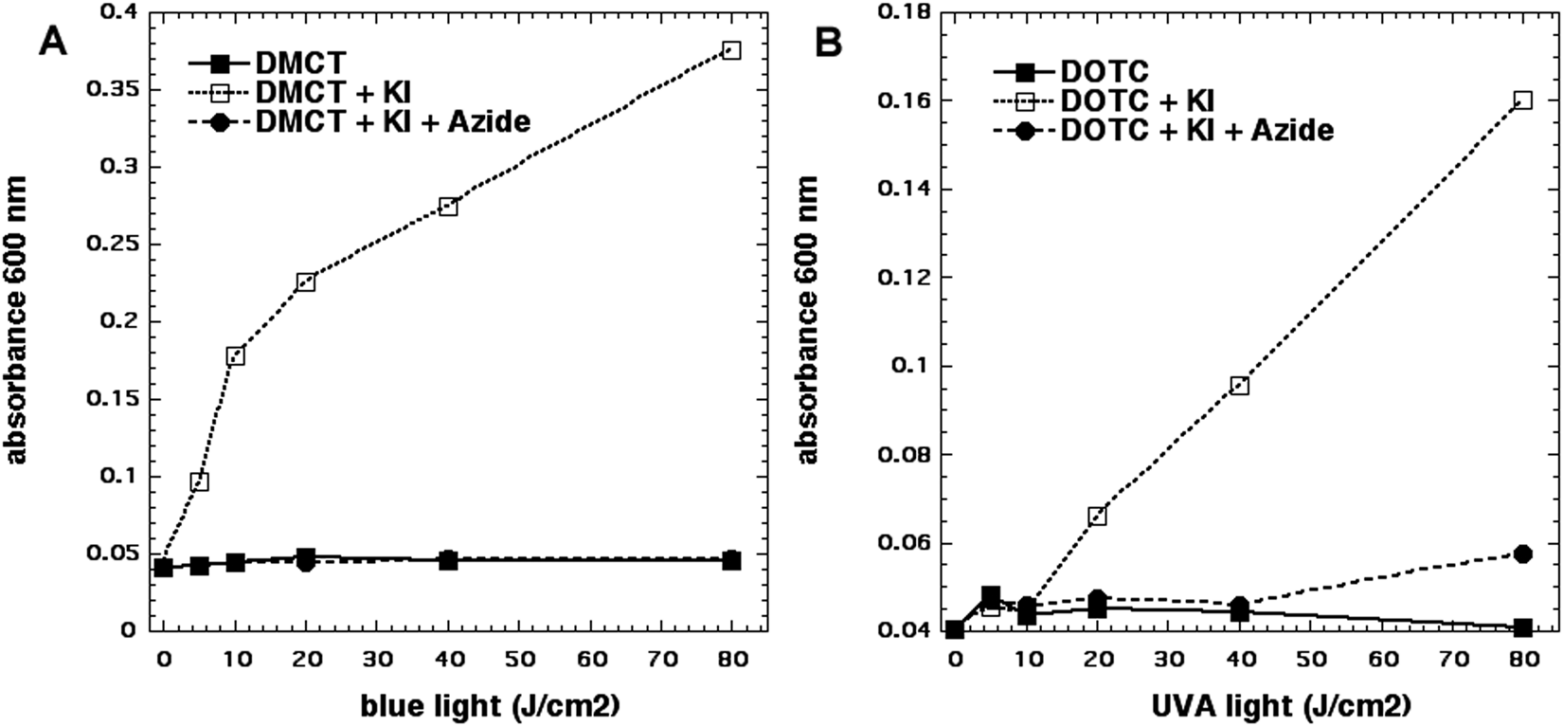
Production of iodine (measured as blue starch complex). Wells contained 50 µM tetracyclines, 400 mM KI, with and without 50 mM azide, excited by 10 J/cm^2^ of UVA or blue light. Aliquots were removed, added to starch indicator and absorbance read at 600 nm.

Since we achieved killing using the “after format”, the obvious candidate to explain this is the formation of molecular iodine (I_2_ or I_3_^−^) as we had previously shown with more conventional PS such as Photofrin^27^, Rose Bengal^28^ or TPPS4^30^. We used the starch assay to quantify the amount of iodine produced by photoexcitation of 50 µM tetracyclines in the presence of 400 mM KI. Moreover, in an attempt to test whether the iodine had been produced by singlet oxygen mediated oxidation of iodide anions we also added 50 mM sodium azide as a singlet oxygen quencher. The results are shown in Fig. 3. In Fig. 3A it can be seen that DMCT excited by blue light produced more than twice as much free iodine as did DOTC excited by UVA light (Fig. 3B). In both cases the production of free iodine was almost completely quenched by addition of azide. It should be mentioned that the production of free iodine by photoexcited tetracyclines was fairly minor when compared to other PS such as Photofrin where more starch color (0.6 OD) was produced with lower concentration of PS (10 µM) and less light (10 J/cm^2^)^27^.

### Production of hydrogen peroxide

**Figure 4:**
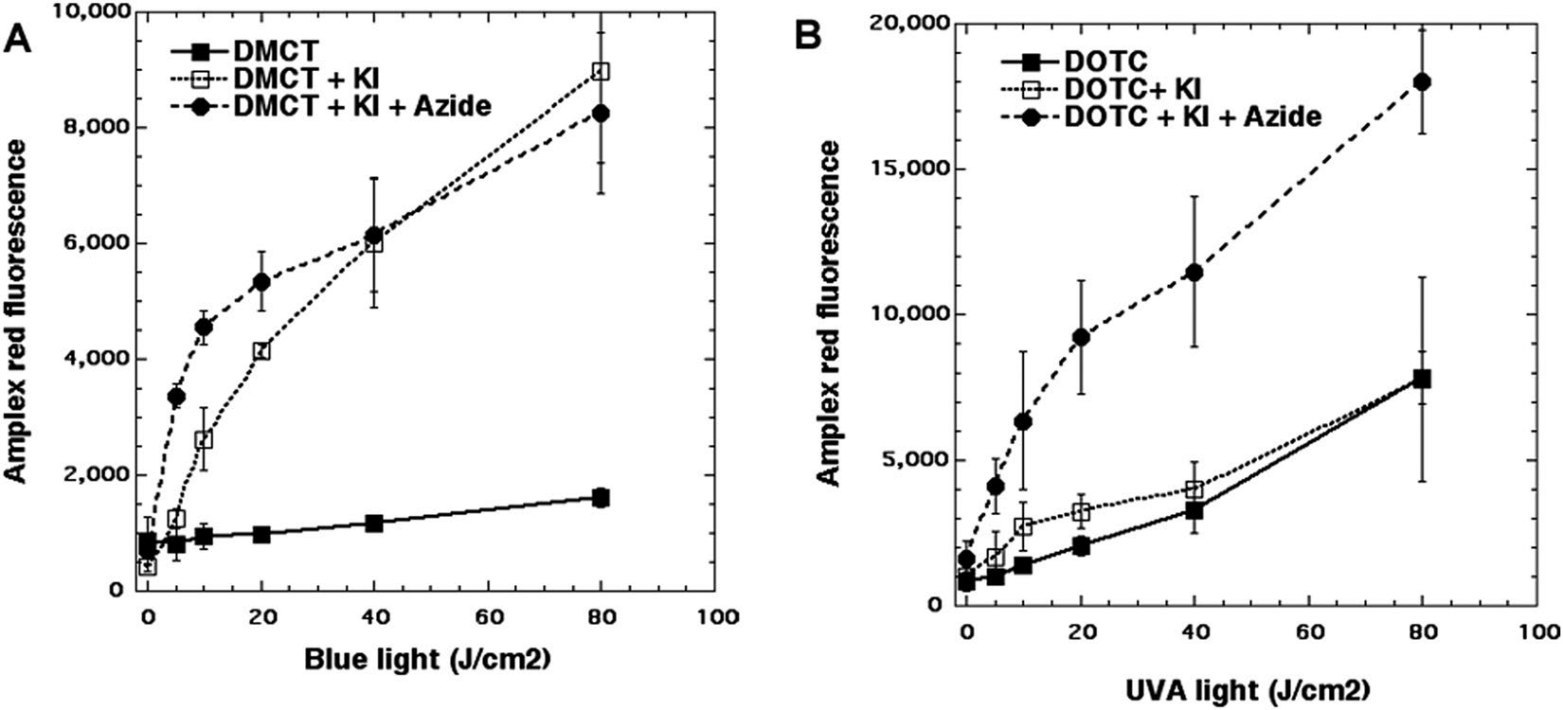
Production of hydrogen peroxide measured by Amplex Red assay. Wells contained 50 µM tetracylines, 400 mM KI, with or without 50 mM azide. Aliquots were withdrawn after each incremental dose of light and added to Amplex Red reagent. (**A**) DMCT excited by blue light; (**B**) DOTC excited by UVA light.

Since the oxidation of KI to free iodine by singlet oxygen also leads to the simultaneous production of hydrogen peroxide we used the Amplex Red assay to measure H_2_O_2_. The results can be seen in Fig. 4. When 50 µM DMCT was excited by blue light there was a modest production of H_2_O_2_ which was increased about 10-fold by the addition of 400 mM KI (Fig. 4A). Interestingly the addition of azide did not quench the production of H_2_O_2,_ and at lower light doses (up to 20 J/cm^2^) azide further increased the H_2_O_2_ to about twice that seen with KI alone. In Fig. 4B we see the analogous results with DOTC excited with UVA light. The baseline production of H_2_O_2_ (i.e. without KI) was about double that seen with DMCT, but the addition of KI made hardly any difference at all. Interestingly the addition of azide produced a large increase in the amount of H_2_O_2_ produced (more than double).

### Activation of ROS probes

**Figure 5:**
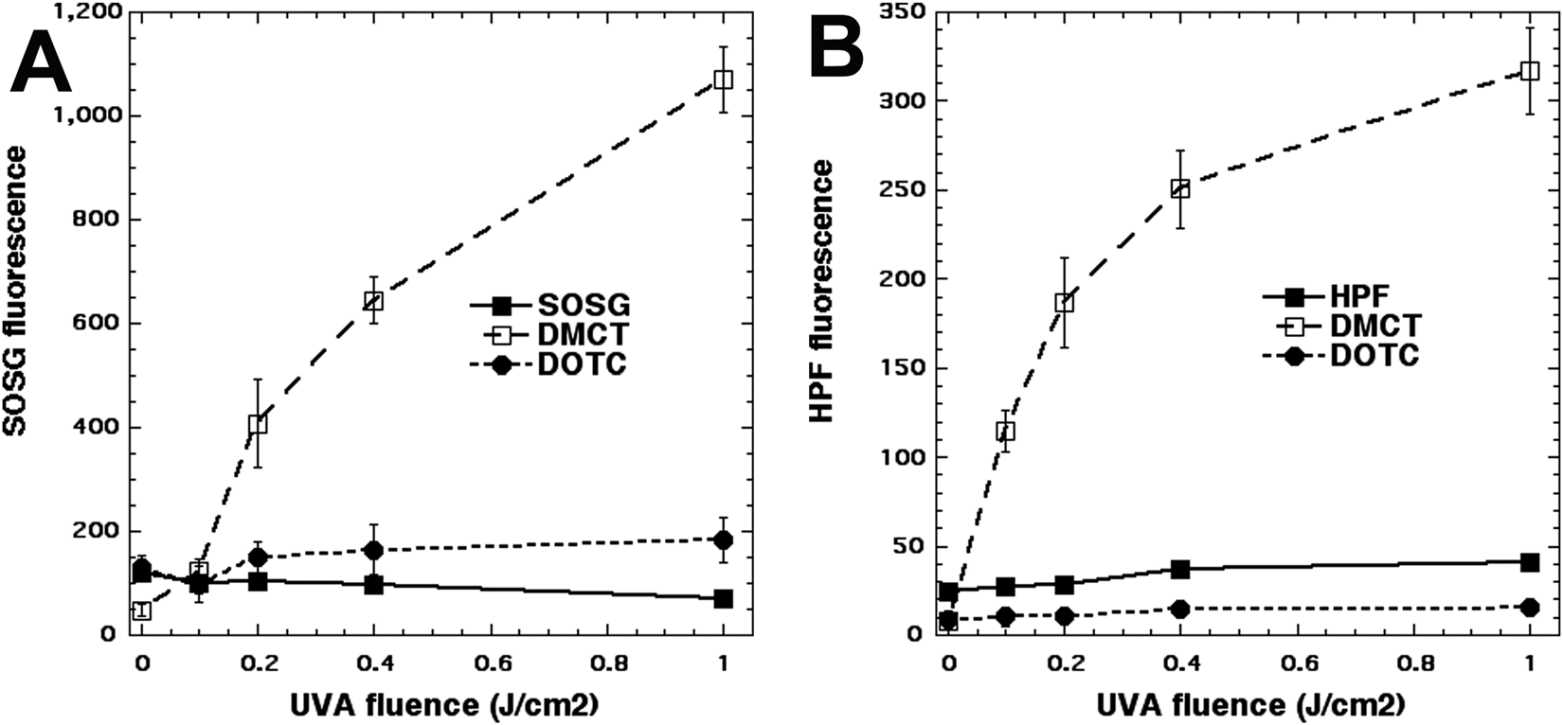
Activation of ROS fluorescent probes by photoexcited tetracyclines. Wells contained tetracyclines 10 µM and probes 10 μM in PBS. Each experimental group contained four wells that were illuminated simultaneously with UVA light in sequential doses of 0–1.0 J/cm^2^.

It was becoming clear that something else was going on with photoactivated tetracyclines beyond the expected Type 1 and Type 2 photochemistry. We used the fluorescent probes for hydroxyl radicals (HPF) and for singlet oxygen (SOSG) to gain some information on the balance between Type 1 and Type 2. We used UVA light to excite both tetracyclines because it has been shown that these probes (particularly SOSG) are activated by blue light alone without any photosensitzer. The results are shown in Fig. 5. Surprisingly it was only DMCT excited by UVA light that activated both probes, HPF in Fig. 5A and SOSG in Fig. 5B. There was almost zero activation of either probe when DOTC was excited by UVA light.

### Iodination of tyrosine

**Figure 6:**
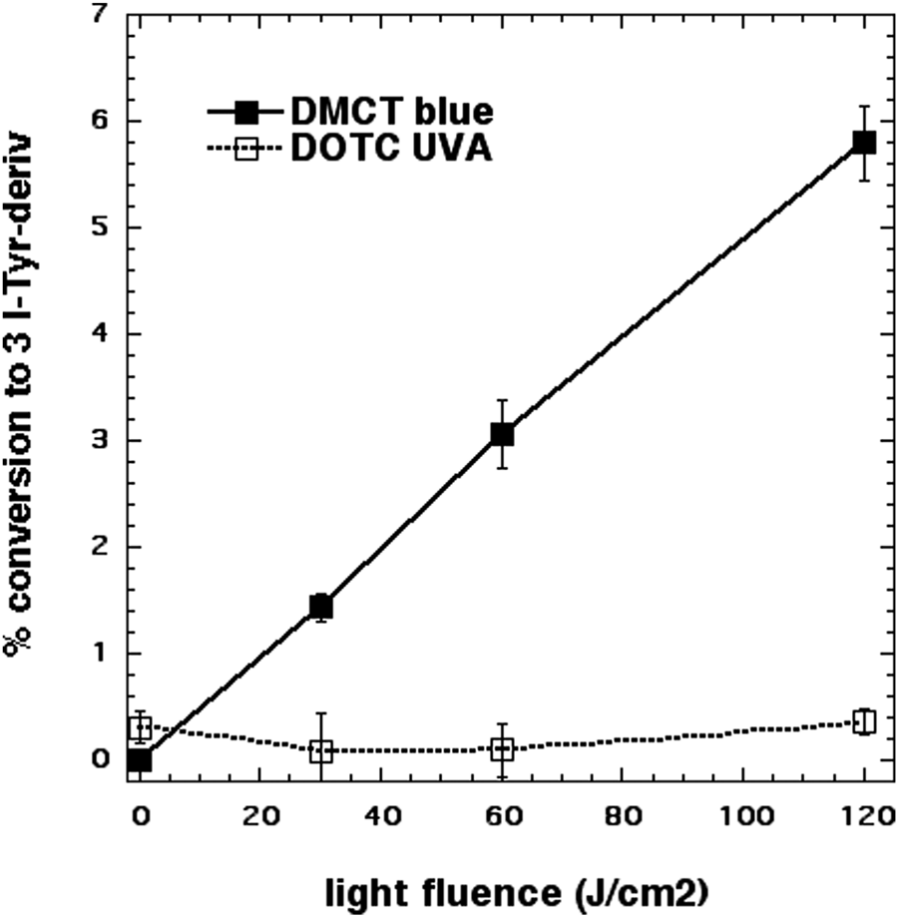
Iodination of tyrosine. Solutions contained 100 µM tetracyclines, 400 mM KI, 10 mM N-acetyl-L-tyrosine ethyl ester and aliquors were removed for KC-MS after aliquots of light had been delivered.

One chemical assay that can be used in experiments where aPDI is potentiated by addition of KI is the iodination of tyrosine. This was carried out using N-acetyl-L-tyrosine ethyl ester as the substrate because the reaction product, N-acetyl-3-iodo-L-tyrosine ethyl ester was readily quantified by LC-MS. Figure 6 shows that although there was a light-dose dependent increase in iodinated tyrosine product mediated by 100 µM DMCT plus 400 mM KI and quite high doses of blue light (up to 120 J/cm^2^), no iodinated tyrosine was detected with 100 µM DOTC (and even with 500 µM DOTC concentration, data not shown) plus 400 mM KI and 120 J cm^2^ UVA light.

### Killing without oxygen and effect of azide

**Figure 7:**
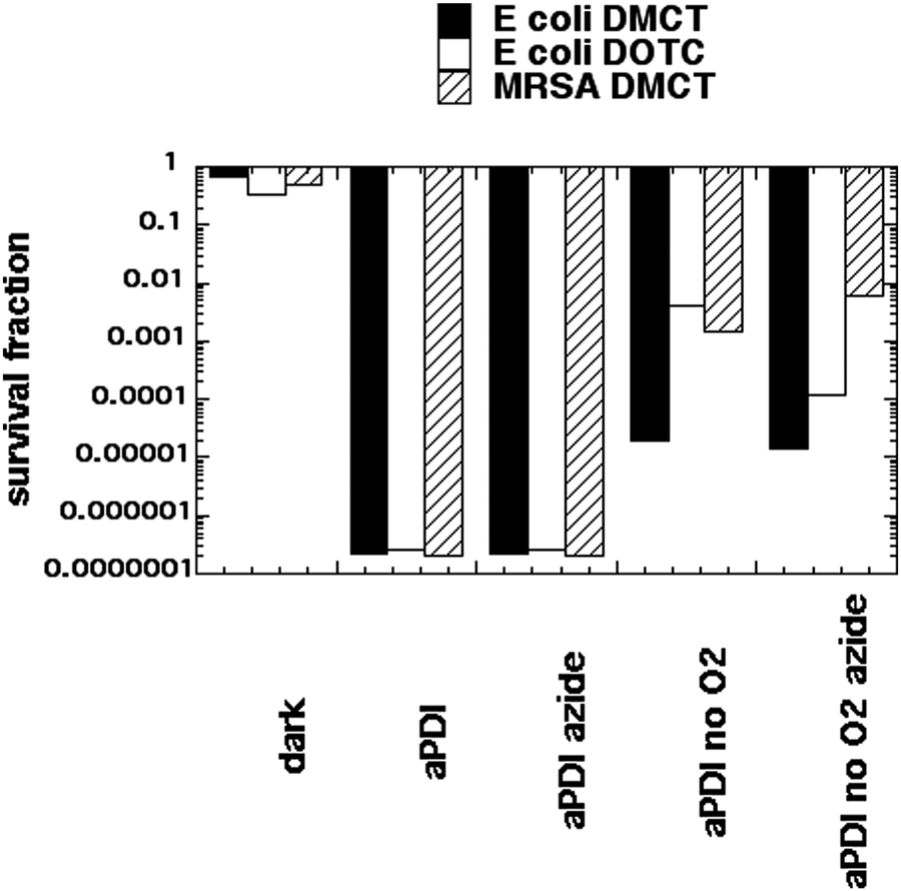
Oxygen independent aPDI and effect of azide. Sealed cuvette contained cells (10(8) CFU/mL. tetracyclines (100 µM), with or without azide (50 mM) and bubbled with N_2_/Ar for 30 min before 10 J/cm^2^ of UVA or blue light was delivered.

When the mechanistic studies presented above were taken into consideration and combined with the data presented in Fig. 2, we formed the hypothesis that the bacterial killing caused by light-activated tetracyclines is mediated by three different mechanisms which may all be operating to some extent at the same time, and these mechanisms would be expected to be affected to very different extents by the addition of KI. These three mechanisms are: (A) Formation of singlet oxygen by a Type 2 process from the triplet state of the tetracycline molecule; (B) Formation of Type 1 ROS (hydrogen peroxide and hydroxyl radicals) from the triplet state of the tetracycline molecule; (C) Direct covalent bond formation between the photoexcited tetracycline (singlet or triplet) and the bacterial ribosomes. It is likely that mechanism A would be the most likely to be strongly potentiated by addition of KI. Mechanism B may also be able to be potentiated by addition of KI. On the other hand, mechanism C is unlikely to be much affected by addition of KI. Moreover, mechanisms A and B will be dependent on the presence of oxygen, but there is a possibility that mechanism C could be oxygen independent. We tested this possibility by comparing aPDI in the presence and absence of oxygen. IN previous studies we had shown that oxygen-independent aPDI could be achieved in the presence of sodium azide (50 mM), when using PS structures that are able to carry out Type 1 photochemical mechanisms (methylene blue^38^ and fullerenes^39^). Therefore, we compared aPDI using a high tetracycline concentration (100 µM) in the presence and absence of air and with and without sodium azide (50 mM). Figure 7 shows the results. At 100 µM concentrations both DMCT activated with 10 J/cm^2^ of blue light and DOTC activated with 10 J/cm^2^ of UVA light eradicated both *E coli* and MRSA (MRSA was only tested with DMCT). When 50 mM azide was added, there was still eradication. When all the oxygen was replaced with nitrogen, we still achieved substantial degrees of killing; 5 logs with *E coli* and DMCT; 2.5 logs with *E*. *coli* and DOTC, and 3 logs with MRSA and DMCT. When 50 mM azide was added in the absence of oxygen the logs of killing remained the same or even increased (DOTC and *E*. *coli*).

### *In vivo* studies

In our previous study^23^ we were not able to carry out any *in vivo* studies using aPDT mediated by tetracyclines. However, the most appealing feature of using tetracyclines as PS, rather than more traditional PS dyes, is the fact that tetracyclines have a dual-antibacterial function. During the illumination period, tetracyclines kill bacteria via the production of reactive species, but during the time following the light delivery, their antibiotic activity will still remain. Therefore, we decided to test *in vivo* PDT in the present study. We decided to use a mouse model of a skin abrasion infected with a pathogenic strain of *E*. *coli* (a UPEC strain that forms biofilms in the bladder)^29^. We chose to test DMCT excited by blue light as this was efficient in killing *E*. *coli* and moreover did not use UV light. In this experiment, we did not explore the possible potentiation of tetracycline-mediated PDT by addition of KI. Although the procedure has been shown to produce major improvements *in vivo* as well as *in vitro*^24,25,28^.

**Figure 8:**
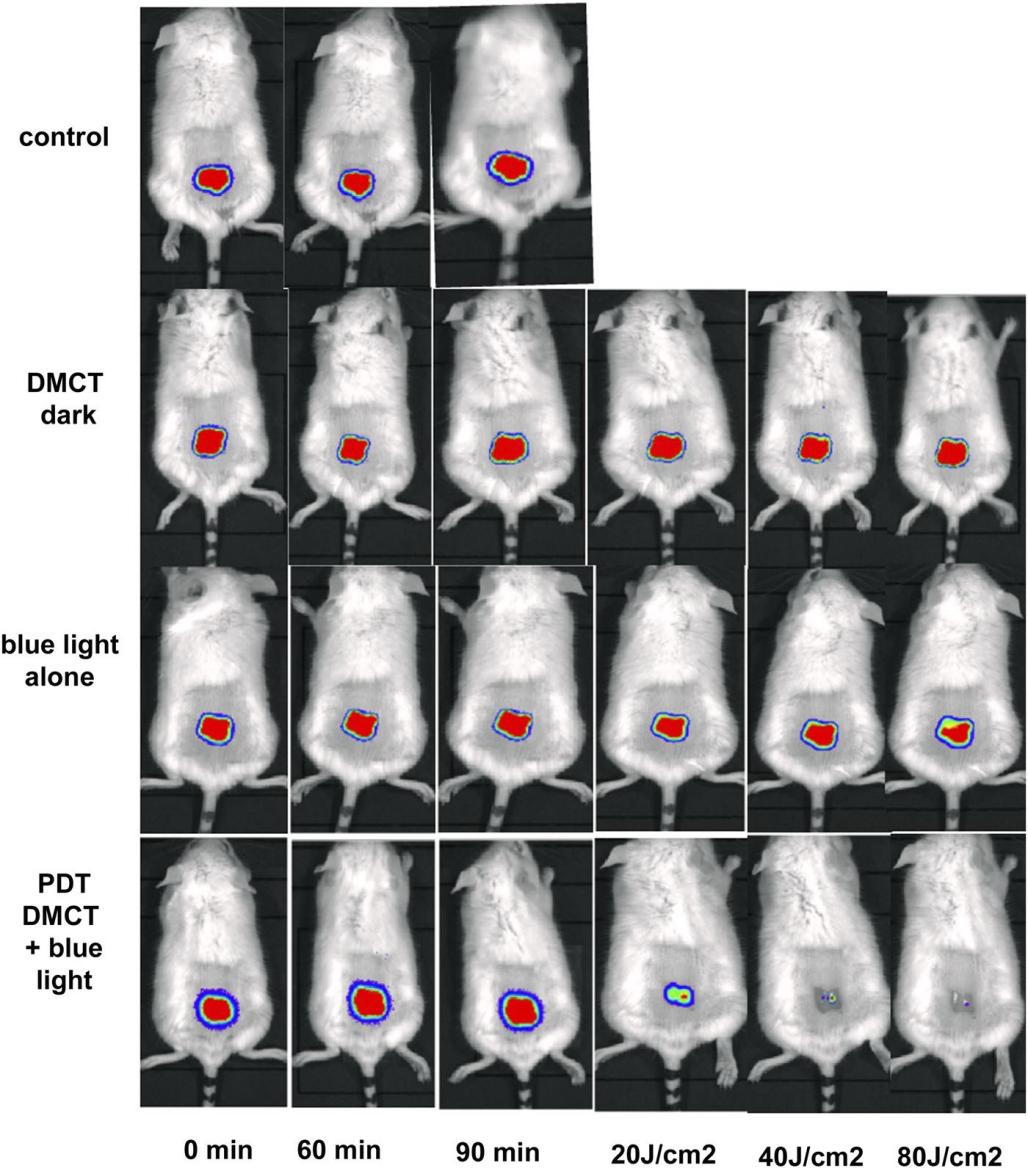
Tetracycline-mediated aPDT of skin abrasions infected with *E*. *coli* monitored by BLI. Representative bioluminescence images from a mouse from each of the treatment groups; no Tx control; 50 µL of DMCT (500 µM) in dark; 50 µL of PBS + blue light; 50 µL of DMCT (500 µM) + blue light. Images were captured after delivery of 0, 20, 40 and 80 J/cm^2^ of 415 nm light (rows 3 and 4) or after equivalent times had elapsed for rows 1 and 2.

**Figure 9:**
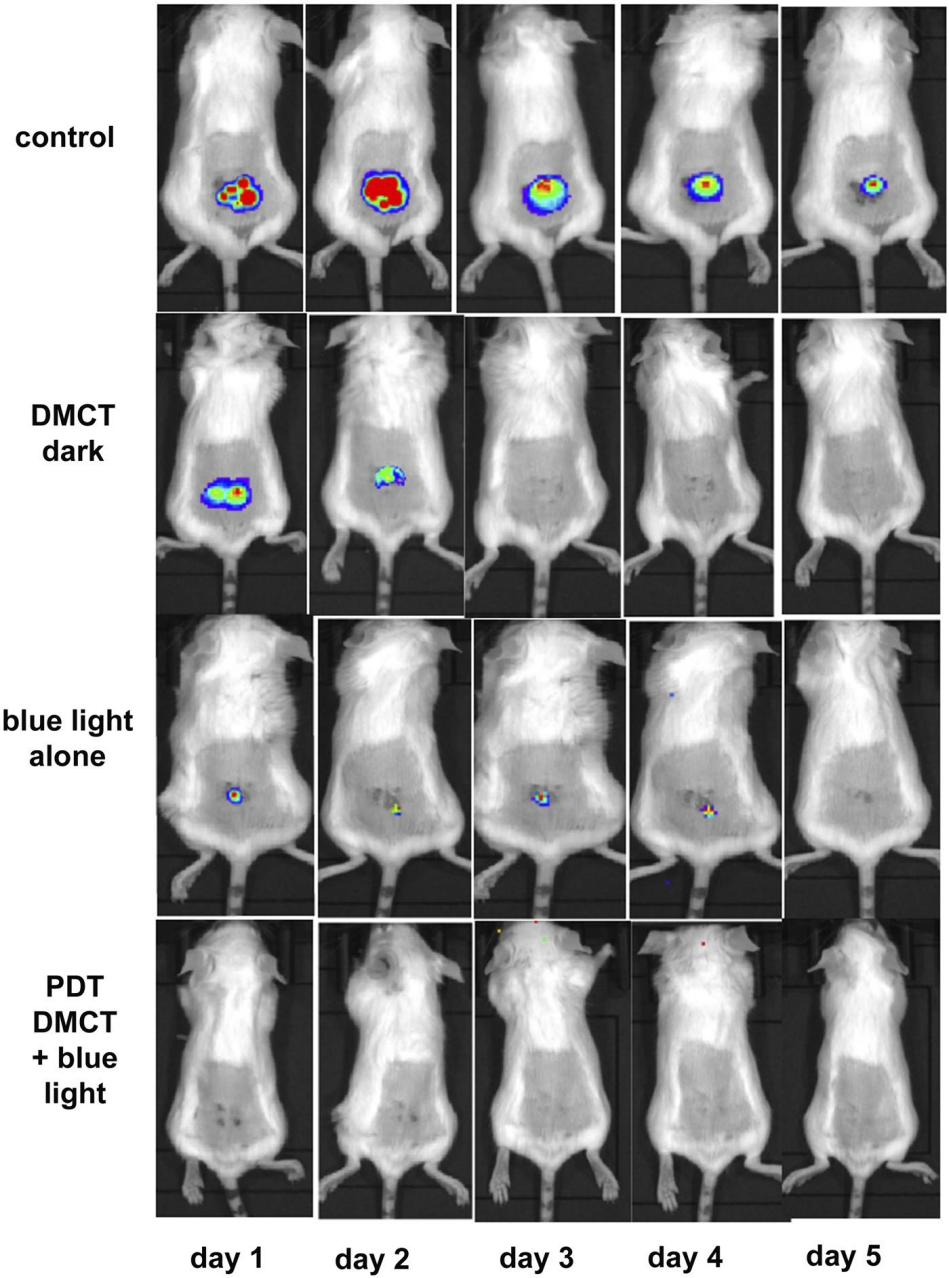
BLI follow-up of aPDT of skin abrasions infected with *E*. *coli* in days following light delivery. Representative bioluminescence images from mice from the 4 groups described in Fig. 8 captured on each day from day 1 after PDT) until day 5.

**Figure 10:**
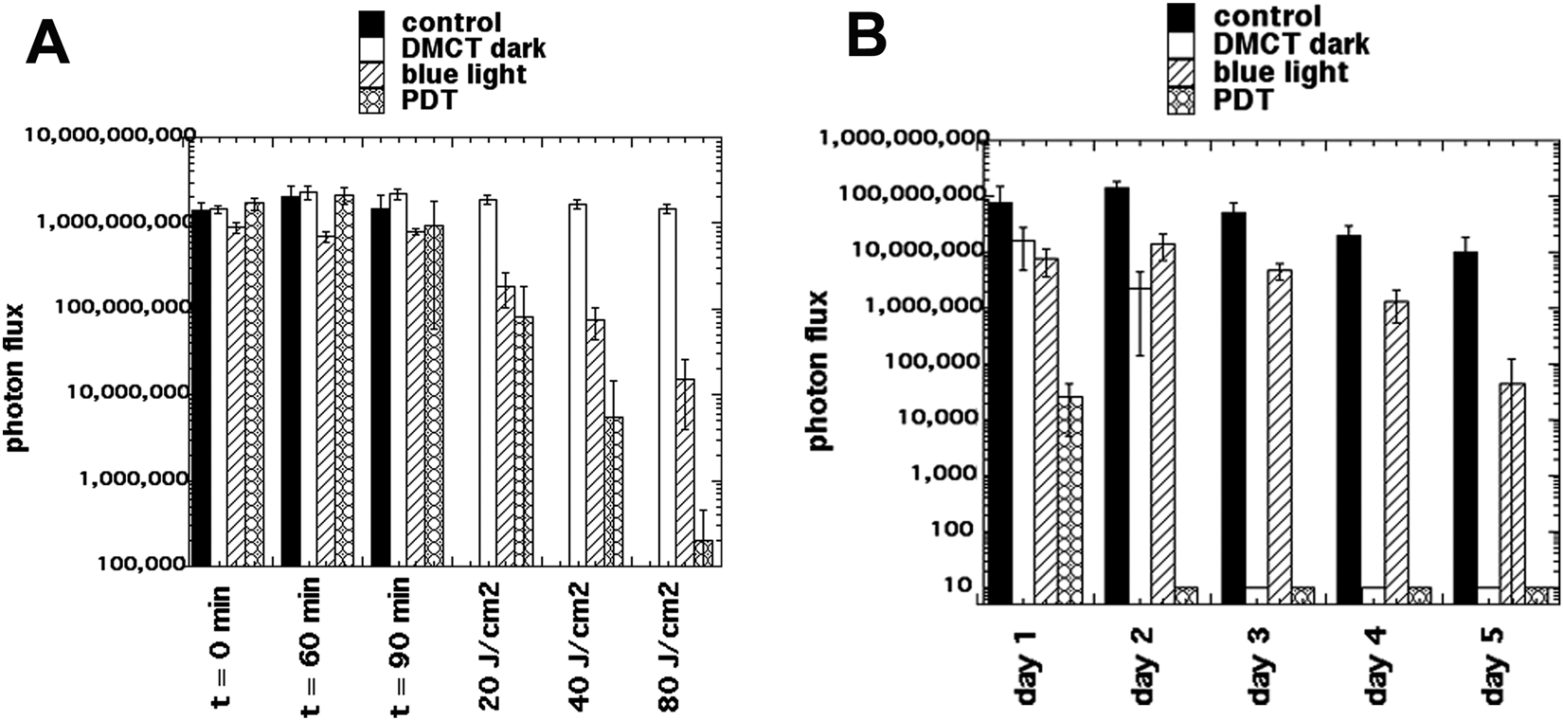
Quantification of bioluminescence signals from the mice in the groups shown in Figs 8 and 9. (**A**) Before and during PDT. (**B**) Over the five days post-PDT. Points are means from (n = 5) mice and bars are SD. *P < 0.05 vs control by one way ANOVA.

Typical representative images from mice in each group are shown in Fig. 8, from the experiment which was carried out on day 0. Images were captured at t = 0 min to ensure equal bacterial loading of wounds, at t = 60 min after bacteria had been allowed to attach to the tissue before the PS was applied, and at t = 90 min after the PS had been allowed to penetrate the bacteria. Topical application of DMCT in the dark did not decrease the bioluminescence signal during the period of the incubation and light delivery (1 hour) but when blue light was delivered at fluences increasing in aliquots up to 80 J/cm^2^, the bioluminescence signal was almost eradicated in a fluence-dependent manner. Blue light alone (at this dosage) did not have a significant effect on the signal. When the mice were followed up over the next five days Fig. 9 the bioluminescence signal remained strongly visible in the control mice, remained at a reduced level in the DMCT dark group for 2 days, remained at a reduced level for 4 days in the blue light alone group, and remained eradicated in the PDT group. The numerical values (mean and SD) of the photon flux density were extracted from all the images using Ivis software and are plotted in Fig. 10A for the PDT experiment and in Fig. 10B for the 5-day follow-up period.

## Discussion

Much of the previous work on the photochemical mechanisms of tetracyclines has been motivated by the clinical observations of skin phototoxicity as a major side-effect of antibiotic therapy with certain tetracyclines. Demeclocycline (90% of patients) and doxycycline (60% of patients) are known to have the highest potential for phototoxicity, while other tetracyclines (minocycline and meclocycline) have not been reported to be phototoxic^40^.

Goldman *et al*.^41^ wanted to determine the whereabouts of the tetracycline binding site(s) inside the bacterial ribosomes. They devised a novel way of doing this using photoaffinity labeling with tetracyclines^42^. They observed photoincorporation of tetracyclines into *E*. *coli* ribosomes occurring via three separate processes: photoincorporation of unaltered tetracycline, photoincorporation of tetracycline photoproduct, and light-independent incorporation of tetracycline photoproduct. Unfortunately, Goldman *et al*. did not investigate whether this photoincorporation process actually killed the bacterial cells. Niu *et al*. showed that tetracycline^43^ was considerably more photostable than found by Hasan^13^. The photobleaching of tetracyclines is probably dependent on the precise wavelength of excitation.

Martin *et al*.^20^ compared the ability of four different tetracyclines (DMCT, DOCT, tetracycline, and oxytetracycline) to carry out aPDI of *E*. *coli* in glucose minimal medium, when excited by UVA light (130 µW/cm^2^ for 70 min). The order of effectiveness was DOCT > DMCT > TC > oxytetracycline. Although the concentrations were higher (up to 160 µM) than we used in the present work, the light fluences were lower. Martin *et al*. used two methods to determine that the photochemical mechanism of action was Type I. Firstly, they used pre-induction of intracellular superoxide dismutase and catalase to inhibit killing. Secondly, they used addition of hydroxyl-radical scavengers^20^. Hasan and Khan^13^ investigated the mechanism of action of tetracyclines in causing skin photoxicity. They reported that it was Type II, involving photosensitized production of “singlet delta dioxygen”. They calculated the relevant singlet oxygen quantum yields to be DMCT = 0.08; TC = 0.05; and MC = 0.00. The lack of singlet oxygen formation by MC was in agreement with our previous finding that MC was essentially without any activity as an antibacterial PS when excited by light^23^. It is presently uncertain which molecular features make DMCT, DOTC and TC photodynamically active, while MC is not.

Our previous study^23^ demonstrated that aPDI using the tetracyclines (DMCT and DOTC) was not only able to kill tetracycline resistant bacterial strains (*E*. *coli*), but moreover that constant illumination was able to significantly reduce the MIC values of both tetracyclines, and this was more pronounced in resistant *E*. *coli* strains.

The present study employed the potentiation of microbial killing by addition of KI to gain mechanistic information on how the photoactivated tetracyclines actually kill bacteria. When we first discovered the KI potentiation effect^24^, we hypothesized that it was mainly a Type 1 process involving an electron transfer reaction with the triplet state PS (Eq. 1) or even with hydroxyl radicals (Eq. 2) to give free iodine which was bactericidal.

1 $${2}^{3}{\rm{PS}}+2{{\rm{I}}}^{-}\to 2{{\rm{PS}}}^{-\bullet }+{{\rm{I}}}_{2}$$

2 $$2{{\rm{HO}}}^{\bullet }+2{{\rm{I}}}^{-}\to 2{{\rm{HO}}}^{-}+{{\rm{I}}}_{2}$$

It subsequently became apparent that singlet oxygen could readily oxidize iodide anion to free iodine and produce hydrogen peroxide^27,28^. The mechanism of this reaction appears to proceed via an initial addition reaction of singlet oxygen to iodide to give iodine hydroperoxide (Eq. 3)

3 $${}^{1}{\rm{O}}_{2}+{{\rm{I}}}^{-}+{{\rm{H}}}_{2}\to {\mathrm{IOOH}+\mathrm{HO}}^{-}$$

The decomposition of peroxyiodide is proposed to proceed via Eqs 4–7

4 $${\rm{IOOH}}+{{\rm{I}}}^{-}\to {{\rm{HOOI}}}_{2}^{-}$$

5 $${{{\rm{HOOI}}}^{-}}_{2}\to {{\rm{I}}}_{2}+{{{\rm{HO}}}^{-}}_{2}$$

6 $${{\rm{I}}}_{2}+{{\rm{I}}}^{-}+\to {{{\rm{I}}}^{-}}_{3}$$

7 $${{{\rm{HO}}}^{-}}_{2}+{{\rm{H}}}_{2}{\rm{O}}\to {{\rm{H}}}_{2}{{\rm{O}}}_{2}+{{\rm{HO}}}^{-}$$

In the present study, we found interesting differences in the photochemical mechanisms between the two tetracyclines. DMCT appeared to be more Type 2 (singlet oxygen) in nature than DOTC, because DMCT produced significantly more free iodine (blue starch color) when KI was added than did DOTC. Moreover, quenching by azide was not entirely complete in the case of DOTC. DMCT showed a large increase in production of H_2_O_2_ (tenfold) when KI was added, and this was not much affected by azide. By contrast DOTC produced a significant amount of H_2_O_2_ when excited by UVA without any KI added, and when KI was added there was hardly any difference in the amount of H_2_O_2._ When azide was added the amount of H_2_O_2_ more than doubled. This interesting observation requires more work to fully understand but may involve intermediate formation of azide radicals and superoxide^44^. When we looked at activation of the fluorescent probes for the ROS, ^1^O_2_ and HO^•^ we found that only DMCT produced significant amounts of probe activation and DOTC did not. We did not show data for probes in the presence of KI, but the activation of both probes was completely quenched by addition of 400 mM KI (data not shown). It should also be noted that the absolute amount of activation of both probes was very small in comparison to levels we have observed with most other PS. If there is a short-lived reactive iodine species produced (for instance iodine radicals (I^•^ or I_2_^•−^) then there is the possibility of iodination of tyrosine. This can be conveniently studied by using the model substrate N-acetyl-L-tyrosine ethyl ester as because the reaction product, N-acetyl-3-iodo-L-tyrosine ethyl ester can be readily quantified by LC-MS. Only DMCT and not DOTC produced measurable amounts of the iodinated reaction product.

It was necessary to use relatively large concentrations of KI (up to 400 mM) to achieve the maximum degree of potentiation. Concerns may be raised that this concentration of KI may be toxic. However it should be remembered that the clinical use of KI solution to treat cutaneous fungal infections, involves the oral administration of saturated solutions of KI in water^45,46^. A saturated solution of KI is over 8 M in concentration.

Taken together our data on photochemical mechanisms combined with reports of photoaffinity labeling of bacterial ribosomes^42^, suggest that the photokilling of bacteria was more complicated than simple aPDI mediated by ROS, and photo-oxidized iodide giving free iodine that acted as a disinfectant. Specifically, the possibility existed of direct and permanent photochemical damage to bacterial ribosomes. Although this damage mechanism could possibly be mediated by generation of ROS, it could also be an oxygen-independent process, in which a covalent bond is formed between the tetracycline molecule itself and amino-acids making up a protein in the 30 S ribosomal subunit^42^. There was a little-known paper from 1969^47^ reporting that isolated *E*. *coli* ribosomes were exceptionally sensitive to photodynamic inactivation by Rose Bengal in the presence of oxygen. The authors attributed this sensitivity to singlet oxygen mediated oxidation of certain amino acid residues in the ribosomal protein as opposed oxidation of the guanine in the RNA. One way for us to investigate the possibility of direct ribosomal photoinactivation was to test whether tetracycline-mediated aPDI could be effective in the absence of oxygen. When we used a comparatively high concentration of both DMCT and DOTC (100 µM) we were able to kill between 3–5 logs of bacteria after 10 J/cm^2^ of light in a nitrogen/argon atmosphere, and moreover the killing was not quenched by azide. We interpret this to mean that the tetracyclines were able to penetrate to the bacterial ribosomes, and covalent bonds were formed after illumination leading to bacterial death, even in the absence of oxygen. Of course, the oxygen-dependent and oxygen-independent mechanisms are both able to operate at the same time in the presence of oxygen. There have been only a few previous demonstrations of oxygen-independent photoinactivation of microbial cells (NB the term “photodynamic” is reserved for those instances where oxygen is definitely involved). A dyad between a porphyrin and a C60 fullerene was able to carry out oxygen-independent photoinactivation due to high levels of photoinduced charge separation^48^. The phenothiazinium dye methylene blue was able to carry out photoinactivation of bacteria in the absence of oxygen, provided 50 mM azide was added to the suspension^38^. Similar results were obtained with two decacationic-functionalized fullerenes, but again only when azide was added^39^. In the latter two cases the mechanism was attributed to the photocatalyzed electron transfer between the excited PS and azide anion, producing azidyl radicals that can damage bacterial cells.

In our previous study^37^ we were not able to test tetracycline-mediated PDT in a mouse model of a wound infection. The compelling advantage of using tetracyclines for *in vivo* PDT against infections, is that after the light is switched off, the antibiotic effect of the remaining non-degraded tetracycline will remain biologically active, and will be able to prevent regrowth of any surviving bacteria remaining after the end of illumination. It is known that in aPDT studies in animal models, the relatively large light doses might lead to photobleaching of the topically applied PS, and additional aliquots of PS might need to be added during the illumination period^49^. However, in the present case there is evidence that the antibiotic activity of the tetracyclines still remained after the light was switched off, so photobleaching cannot have been complete. This is not the case with the vast majority of antimicrobial PS, where the killing effect is finished as soon as the light is switched off. We were able to demonstrate this advantage in a mouse model of a superficial wound abrasion infected with a bioluminescent Gram-negative bacterial species. There was no recurrence of bioluminescence in the five days after PDT. This can be contrasted with PDT studies in similar animal models of superficial infections using traditional type PS where substantial recurrence of bioluminescence signal in the succeeding days was definitely observed^50–52^. There are further experiments that should be carried out to confirm the findings of the present study. The *in vivo* animal experiments should be repeated using DOTC and UVA light. This is necessary because a considerable amount of hydrogen peroxide was also produced in case of DOTC + KI. The *in vivo* experiments should be carried to test whether addition of KI can also potentiate the PDT effect *in vivo*. However we did previously show that KI could effectively potentiate the *in vivo* PDT mediated by Rose Bengal in a mouse model of skin abrasions infected with bioluminescent *Pseudomonas aeruginosa*^28^. Moreover we also showed that addition of KI could also potentiate aPDT mediated by functionalized fullerenes, in the same mouse model of skin abrasions infected by bioluminescent *Acinetobacter baumannii*^25^. Therefore we would expect that the same potentiation would occur *in vivo* with tetracycline PDT plus added KI.

In conclusion DMCT and DOTC are dual-action antibacterial compounds functioning as antibiotics in the dark and as PS under illumination with blue or UVA light. Their photochemical mechanisms are complex and include Type 2 generation of singlet oxygen, Type 1 generation of radical intermediates, and a direct oxygen-independent photoaffinity labeling of ribosomes. Their aPDI activity can be strongly potentiated (up of 5 extra logs of killing) by addition of KI in the 200–400 mM range. Blue light activation could be considered clinically in certain situations where tetracyclines are routinely used for infections that are accessible to light delivery. Moreover, topical addition of KI could also be tested clinically as even high concentrations (saturated solution of 8 M) are non-toxic.

## Data Availability

Original data are deposited. https://dataverse.harvard.edu/dataset.xhtml?persistentId=doi%3A10.7910%2FDVN%2FPAEMNT&version=DRAFT.
